# Cholecalciferol and Cancer: Is It a Big D_3_-eal?

**DOI:** 10.6004/jadpro.2012.3.4.6

**Published:** 2012-07-01

**Authors:** Rita Wickham

**Affiliations:** From Northern Michigan University, Marquette, Michigan

Although the associations between cholecalciferol (hereafter referred to as vitamin D) and health are only beginning to be deciphered, we have adequate understanding to apply some concepts to the care of individuals with cancer. Vitamin D, which is synthesized or ingested by every living organism, is essential to the function of all cells to maintain extracellular and intracellular calcium homeostasis, cell signaling, and regulation of numerous physiologic functions (Bikle, 2010).

The optimal amount of vitamin D humans need each day remains controversial, particularly in light of the current recommended daily intake (RDI) from the Institute of Medicine (IOM, 2011). This is an important consideration because 50% to 75% of Caucasians and as many as 90% of African Americans, Asians, and Latinos in the United States are vitamin D insufficient or deficient (Armas et al., 2007; Adams & Hewison, 2010; Binkley, Ramamurthy, & Krueger, 2010; Kennel, Drake, & Hurley, 2010; Wimalawansa, 2012). These estimates are similar or worse in cancer patients or survivors; in one study of 413 women about to begin aromatase inhibitor (AI) therapy for breast cancer, only 13% had normal vitamin D levels before they started therapy (Singh, Cuzick, Mesher, Richmond, & Howell, 2012). This may have other clinical consequences as well. For instance, the authors of a recent extensive review concluded that the low nutritional vitamin D status of African Americans may explain the unaccounted for disparity in cancer survival rates between African Americans and White Americans (Grant & Peiris, 2012).

Low vitamin D has also been documented in some patients with other cancers, cardiovascular disease, autoimmune (e.g., rheumatoid arthritis and multiple sclerosis) or infectious conditions, osteoporosis, type 2 diabetes, and obesity and may increase the risk for cognitive impairment in elderly individuals (Grey & Bolland, 2010; Hanley, Cranney, Jones, Whiting, & Leslie, 2010; Pearce & Cheetham, 2010). Most studies that examined such relationships were observational, so their strength and direction remain to be established in prospective trials. It is not known whether vitamin D deficiency and disease reflects a cause-and-effect relationship, or if the deficiency is a manifestation common to poor health.

A hot-off-the-presses meta-analysis of randomized clinical trials examined the effect of vitamin D alone or with calcium on mortality in 88,097 elderly individuals (Rejnmark et al., 2012). The major study finding was that vitamin D (10-20 ìg [400-800 IU] per day) plus calcium (but not vitamin D alone) reduces mortality (absolute risk reduction, 0.66).

## What Is Vitamin D and Why Do We Need It?

The “discovery” of vitamin D was driven by the high incidence of rickets during the Industrial Age, heralded by increased smog, decreased exposure to sunlight, and child labor. The hallmarks of rickets are painful or tender soft bones, short stature, muscle weakness, bone and teeth deformities, and reproductive problems secondary to skeletal defects. Around 1920, two discoveries were made: A factor that was identified in cod liver oil and named vitamin D (discovered after vitamins A, B, and C) could prevent rickets in dogs, and ultraviolet light could cure rickets in children (DeLuca, 2008).

We now know that vitamin D, often in conjunction with calcium, is involved in numerous body functions. Vitamin D promotes calcium absorption in the gut, maintains the bone-calcium hydroxyapatite structure critical to bone mineral density, is necessary for muscle function, maintains normal serum calcium and phosphate levels, and is involved in many cellular functions. Despite all of these functions, the current IOM RDIs address only the amount of vitamin D that will prevent rickets in children and osteomalacia in adults. Some scientists and clinicians disagree with the IOM recommendations because the optimal serum levels of vitamin D metabolites for other essential roles are unknown; they propose measuring nutritional vitamin D (calcidiol) to determine individualized supplementation needs (Heaney & Holick, 2011; Hollis, 2011). Similarly, the Endocrine Society recently published consensus-based guidelines regarding vitamin D (Holick et al., 2011), which are meant to help clinicians identify and evaluate patients at risk for vitamin D deficiency, as well as to guide efforts in prevention (see Table 1).

**Table 1:**
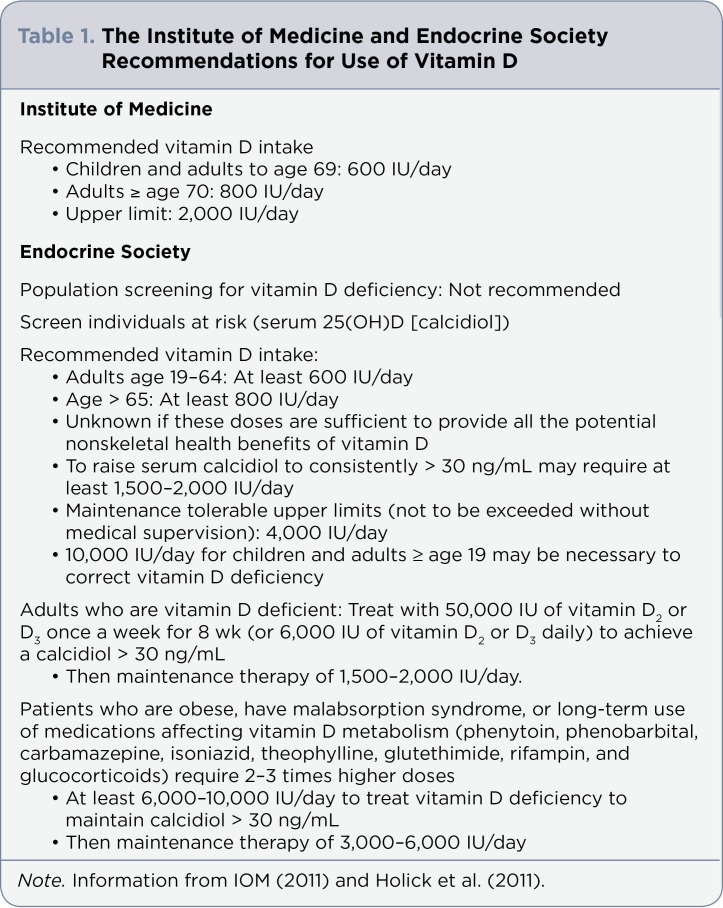
Table 1. The Institute of Medicine and Endocrine Society Recommendations for Use of Vitamin D

If it were identified today, vitamin D would not be called a vitamin—a vital nutrient that must be obtained in small amounts from the diet—but more correctly a prohormone essential to life. It is metabolized via two steps to a short-lived, biologically active pluripotent steroid hormone with numerous endocrine and paracrine functions (Norman, 2008). Vitamin D is highly conserved and found in virtually all plants and animals, even deep-ocean single-cell phytoplankton and zooplankton that have different mechanisms of production (Bikle, 2010). Animals synthesize D_3_ (cholecalciferol), whereas most plants and fungi synthesize D_2_ (ergocalciferol); these have slightly different chemical structures but are similarly metabolized.

**Figure 1:**
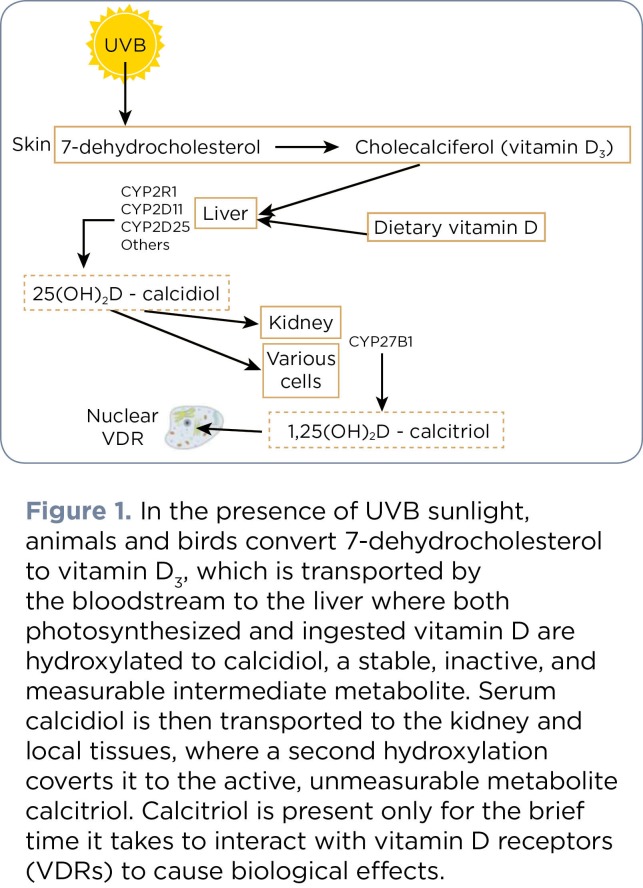
Figure 1. In the presence of UVB sunlight, animals and birds convert 7-dehydrocholesterol to vitamin D_3_, which is transported by the bloodstream to the liver where both photosynthesized and ingested vitamin D are hydroxylated to calcidiol, a stable, inactive, and measurable intermediate metabolite. Serum calcidiol is then transported to the kidney and local tissues, where a second hydroxylation coverts it to the active, unmeasurable metabolite calcitriol. Calcitriol is present only for the brief time it takes to interact with vitamin D receptors (VDRs) to cause biological effects.

As shown in Figure 1, after synthesis or ingestion, vitamin D is transported to the liver, where cytochrome P450 enzymes (CYP2R1, CYP2D11, and CYP2D25) hydroxylate it to an intermediate, measurable metabolite: 25(OH)D (calcidiol). Serum calcidiol is subsequently transported to the kidney, where parathyroid hormone (PTH) regulates mitochondrial CYP27B1 hydroxylation to the short-lived, active vitamin D metabolite: 1,25(OH)2D (calcitriol). When circulating calcidiol is sufficient, many cells (e.g., skin, brain, testes, intestine, lymph nodes, bone, cartilage, and placenta) elaborate CYP27B1 and directly synthesize calcitriol when needed for hormone or cytokine effects. These include monocytes and macrophages, in which calcitriol acts as a cytokine to modulate innate immunity (Adams & Hewison, 2010).

Almost all cells express nuclear vitamin D receptors that mediate local actions of calcitriol and regulate the expression of more than 200 genes. These include adipose, adrenal, bone, bone marrow, brain, breast, colon, intestine, kidney, lung, B and T lymphocytes, cardiac and smooth muscle, ovarian, pancreatic beta cells, parathyroid, pituitary, prostate, skin, stomach, testis, thyroid, and uterine, as well as some cancer cells (Norman, 2008). Calcitriol is a powerful regulator of cellular growth in normal and cancer cells; it inhibits cellular proliferation and induces terminal differentiation, inhibits angiogenesis, stimulates insulin production, inhibits renin production, stimulates macrophage cathelicidin production, and stimulates its own destruction (Holick et al., 2011; Raisz, 2005; Stechschulte, Kirsner, & Federman, 2009). In bone, calcitriol, along with PTH and IL-6, indirectly increases the receptor activator for nuclear ĸB ligand (Murthy et al., 2010).

On June 19, 2012, at the American Association for Cancer Research meeting focusing on pancreatic cancer, a presentation summarized the findings from two large studies in which a single nucleotide polymorphism (SNP, pronounced “snip”) in the VDR gene called rs2853564 was related to survival in patients with advanced pancreatic cancer. Median overall survival was longest in patients homozygous for rs2853564 (inherited this allele from both parents), 8.9 to 10.5 months; shorter in heterozygous patients (inherited the rs2853564 allele from one parent and a different allele from the other parent), 5.9 to 8.3 months; and shortest in patients without an rs2853564 allele, 4.7 to 6.6 months (Innocenti et al., 2012). Confirmation of these data could spur renewed interest in vitamin D analogs as anticancer therapy.

## Why Is Vitamin D Deficiency So Common?

In past times, humans got 90% of their vitamin D from ultraviolet B (UVB) sunlight and about 10% from their diet. A “good diet” cannot prevent deficiency because few foods naturally contain vitamin D: mainly wild-caught oily ocean fish and egg yolks (Kennel et al., 2010). Consuming these and fortified foods results in a dietary vitamin D intake of about 150–200 IU (3.75–5 ìg) per day; see Table 2 for a list of foods and how much vitamin D they contain (Bailey et al., 2010; Holick & Chen, 2008; Moyad, 2008).

**Table 2:**
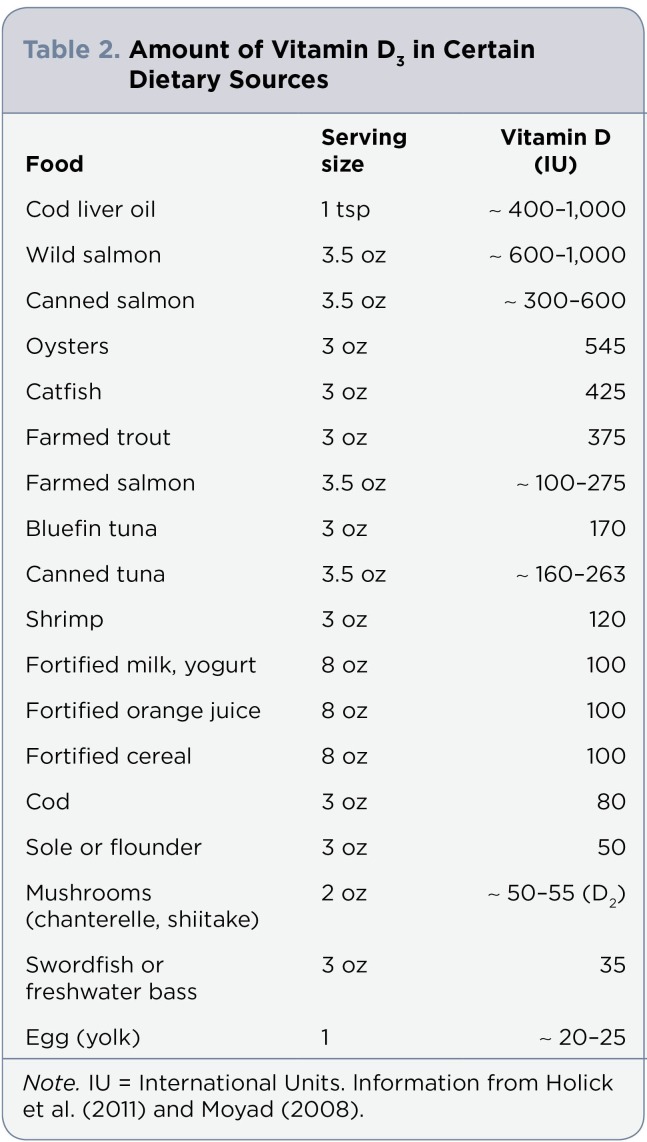
Table 2. Amount of Vitamin D_3_ in Certain Dietary Sources

**Figure 2:**
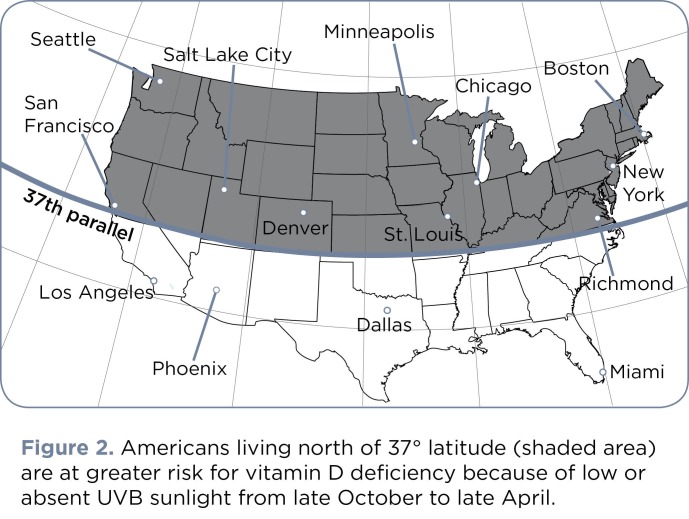
Figure 2. Americans living north of 37° latitude (shaded area) are at greater risk for vitamin D deficiency because of low or absent UVB sunlight from late October to late April.

UVB sunlight causes photoconversion of 7-dehydrocholesterol in the skin to cholecalciferol (vitamin D_3_). In much of the United States (north of 37° latitude), synthesis varies depending on the season (highest in summer and lowest in winter) and the time of the day (UVB sunlight occurs from 10 a.m. to 3 p.m. from late spring to early fall; see Figure 2). Other factors that decrease vitamin D synthesis are wearing clothing or using sunscreen to block the sun’s rays, staying out of the sun all together, aging (decreased dehydrocholesterol in skin lessens synthesis), and having darker skin (melanin blocks the sun’s rays) (Armas et al., 2007).

Educational campaigns about the dangers of sun exposure with regard to skin cancers have dramatically increased vitamin D deficiency worldwide. Public health campaigns that espouse total sun avoidance do not take into account how humans normally synthesize vitamin D. As humans evolved in equatorial Africa, they lost body hair and developed sweat glands for cooling. Because the levels of UVB (and UVA) sunlight are highest at the equator, synthesizing vitamin D year round was not an issue. Melanin expression and dark skin are thought to have evolved to conserve folate necessary for reproduction. This was less necessary in groups of humans who migrated away from the equator and eventually came to have lighter skin that would not block as much UVB sunlight and more efficiently synthesize vitamin D_3_ (Jablonski & Chaplin, 2002).

Recent increases in mobility and large-scale migration, along with sun avoidance, have led to the pandemic of vitamin D deficiency, particularly in darker-skinned persons. A reasonable rule of thumb for advising most patients about sensible sun exposure during the hours of 10 a.m. to 3 p.m. during late spring, summer, and autumn is that they should apply sunscreen after the time it takes to cause slight skin erythema at 24 hours (1 minimal erythemal dose [MED]): about 15 minutes for fair-skinned people, 30 minutes for Asian Indians, and up to 120 minutes for African Americans (Hollis, 2005).

In 1 MED, an adult wearing a swimsuit would synthesize about 10,000 to 25,000 IU of vitamin D_3_ (Holick, 2010). After 1 MED, no more cutaneous vitamin D_3 _ synthesis occurs, but the risks for photoaging and skin cancers increase. So you can also advise your patients always to use sunscreen of at least SPF 15 on their face and hands, which will almost totally block cutaneous synthesis of vitamin D_3_, and to apply it to other exposed body parts after reaching their MED time (Armas et al., 2007; Sayre & Dowdy, 2007).

## How Much Vitamin D Is Needed?

The solution for most individuals is vitamin D supplementation. Both ergocalciferol (D_2_) and cholecalciferol (D_3_) oral supplements are available (prescription and over-the-counter, respectively). D_2_ and D_3_ are equivalent when administered daily or weekly, but D_3_ has a longer half-life and greater affinity for D-binding proteins than does D_2_ and leads to more consistent levels when administered at longer intervals (Wimalawansa, 2012).

The most widely publicized recommendations are from the IOM (2011); they are assumed to result in nutritional vitamin D (calcidiol) levels sufficient to prevent rickets in children, osteomalacia in adults, and falls in the elderly. Given the high incidence of vitamin D deficiency, the IOM RDIs may not be sufficient to increase or maintain “normal” or “optimal” vitamin D levels, which are more accurately measured by circulating serum calcidiol status. On the other hand, the Endocrine Society recommends similar RDIs for the general population but also advises screening for vitamin D deficiency, which certainly includes many patients with cancer, by checking calcidiol levels and more aggressively managing these patients; see Table 1 (Hollis, 2011).

There are no universally agreed-upon definitions for overall normal and deficient calcidiol values. A level of ≤ 20 ng/mL (or 50 nmol/L) is considered “deficiency” and can lead to impaired bone mineralization, with rickets in children or osteomalacia in adults (Binkley, Ramamurthy, & Krueger, 2010; Grey & Bolland, 2010; Khan et al., 2010; IOM, 2011); a level of 21–31 ng/mL (52.5–77.5 nmol/L) is considered “insufficiency” and may be associated with long-term adverse health and bone effects; and 32–100 ng/mL (80–250 nmol/L) is the “normal” calcidiol range.

However, previous methods to establish normal calcidiol levels may have been flawed. Hollis (2005) proposed that it would make more sense to consider levels from 54–90 ng/mL (in persons who work outside without being overly clothed) as normal and levels < 32 ng/mL as deficient. In the same vein, we do not have a way to establish normal population vitamin D levels (Wimalawansa, 2012). These are important considerations, particularly because most studies involving the health effects of vitamin D have been retrospective, no standard doses of vitamin D have been used, serum calcidiol levels were frequently not assessed, and current definitions of recommended and upper limit doses may or may not reflect actual effects.

## Potential Problems Related to Vitamin D Deficiency

The vitamin D deficiency literature is growing at a phenomenal rate. In terms of cancer patients or survivors, this means we are reading more about potential relationships of vitamin D deficiency and aromatase inhibitor–induced musculoskeletal symptoms (AIMSS) as well as cancer and cancer mortality, impaired bone health (osteoporosis or osteomalacia), and adverse effects with administration of bisphosphonates or denosumab (Prolia, Xgeva). Vitamin D deficiency may be accompanied by subtle and nonspecific manifestations: generalized bone discomfort possibly elicited with pressure on the sternum or tibia, lethargy, worsening of chronic disease, rheumatoid arthritis, muscle aches, osteoporosis, low back pain (in women), proximal muscle weakness and increased risk for falls, difficulty losing weight, or proximal myopathy potentially misdiagnosed as fibromyalgia, chronic fatigue syndrome, or arthritis (Bordelon, Ghetu, & Langan, 2009; Wimalawansa, 2012).

A comprehensive discussion of vitamin D deficiency and cancer occurrence and mortality is beyond the scope of this article. Interest has been spurred by ecologic studies that noted the incidence and prevalence of some cancers increase with greater distance from the equator and that cancer survival is better for individuals diagnosed in summer months when UVB sunlight is available (Bell, 2011; Krishnan, Trump, Johnson, & Feldman, 2010). The possible connection between vitamin D and cancer is hypothesized because vitamin D has antiproliferative and differentiation roles, inhibits telomerase expression and angiogenesis, and induces apoptosis. Vitamin D may also exert anticancer effects by influencing gene transcription and cell signaling, have other cell cycle effects (Fleet, Desmet, Johnson, & Li, 2012), and may also reduce invasion and metastases (Krishnan et al., 2010).

A recent meta-analysis of prospective randomized and observational studies suggested that high-dose vitamin D (1,000 IU/day) can decrease the total risk for cancer and that each 4 ng/mL increase in serum calcidiol decreases the risk for colorectal cancer (but not breast or prostate cancer) by 6% (Chung, Lee, Terasawa, Lau, & Trikalinos, 2011). Calcidiol levels as high as 60–80 ng/mL may be needed to reduce cancer risk (Garland, French, Baggerly, & Heaney, 2011).

As discussed on page 245 by Jeannine Brant, PhD, APRN, AOCN®, advanced practitioners (APs) frequently encounter AIMSS. Somewhere between 18% and 45% of breast cancer patients develop AIMSS, usually within 2 months of starting AI therapy. Manifestations include bilateral joint pain and stiffness (hands, shoulders, lower back, hips, knees, and feet) and possible morning stiffness, sleep problems, fatigue, and functional impairment (Burstein, 2007; Khan et al., 2010; Winters, Habin, Flanagan, & Cashavelly, 2009). Unrelieved pain is the most common reason for discontinuing a prescribed AI (Dent, Gaspo, Kissner, & Pritchard, 2011), and symptoms may be worst in young women (ages 35–50) with ovarian failure secondary to chemotherapy or surgery (Al-Janadi et al., 2010).

Unrecognized vitamin D deficiency can also cause painful osteomalacia that may be misdiagnosed as bone metastases on x-ray (Khokhar, Brett, & Desai, 2009). Another serious complication can occur after bisphosphonate administration (e.g., zoledronic acid or pamidronate) to vitamin D–deficient patients: severe and potentially life-threatening hypocalcemia (reported levels 5.2–9 mg/dL to 6.9 mg/dL) accompanied by cardiac abnormalities and with elevated (1.2–2.6 mg/dL) serum creatinine levels (Breen & Shane, 2004; Broadbent, Glare, & Crawford, 2005; Rosen & Brown, 2003; Wang-Gillam, Miles, & Hutchins, 2008). Hypocalcemia can also occur with denosumab administration, but there is no information on whether vitamin D deficiency increases risk (Henry et al., 2011).

## Implications for Advanced Practitioners

When AI therapy is planned, the advanced practictioner should have an in-depth discussion with patients regarding possible side effects as well as benefits of therapy, remind them about the importance of adherence and possible interventions if AIMSS occurs, and document baseline musculoskeletal symptoms. A baseline serum calcidiol level may be indicated because many breast cancer patients are vitamin D deficient, which may be a factor for worsening AIMSS.

Two prospective studies examined the effects of vitamin D levels and supplementation on AIMSS-related arthralgia. One study stratified 60 breast cancer patients about to begin an AI by calcidiol levels: Those with levels ≤ 40 ng/mL were assigned to high-dose vitamin D supplementation of 50,000 IU oral vitamin D every week for 12 weeks, and those > 40 ng/mL continued 600 IU/day (Khan et al., 2010). Sixteen weeks after starting an AI, women whose calcidiol was > 66 ng/mL were significantly more likely to have less pain and no disability from AIMSS than were women with calcidiol levels < 66 ng/mL (*p* = .008).

In a second prospective study, Prieto-Alhambra et al. (2011) identified 260 postmenopausal breast cancer patients about to begin AI therapy who had calcidiol levels of < 30 ng/mL. They were treated with oral calcium (1 g) and vitamin D (800 IU) daily plus oral vitamin D 16,000 IU every 2 weeks for 3 months. At the end of this study period, women whose calcidiol was > 40 ng/mL were less likely to develop joint pain than those with levels of < 40 ng/mL (*p* = .008). However, only about half of these patients treated with high doses of vitamin D used in this study (cumulative dose = 280,000 IU) had a serum calcidiol of > 30 ng/mL at the end of the study. These two studies illustrate that vitamin D may be helpful in alleviating AIMSS, but vitamin D deficiency is a significant problem, and patients with breast cancer receiving an AI may require higher doses of vitamin D and more frequent monitoring to achieve a level of > 40–65 ng/mL.

Some patients may be reluctant to start or continue vitamin D because of recent reports in the lay literature that the United States Preventive Services Task Force has recommended that women should not take vitamin D, with or without calcium, to prevent osteoporotic fractures or cancer (Kolata, 2012). Patients can be reminded that this is a draft recommendation that has been posted for public comment, and that their reason for taking vitamin D is different.

Anecdotal reports suggest that AIMSS may be lessened or controlled with analgesics (a nonsteroidal anti-inflammatory drug, a COX-2 inhibitor, tramadol, or an opioid), or another agent (e.g., glucosamine plus chondroitin sulfate, an anticonvulsant, or topical methylsalicylate) may be effective for many patients (Crew et al., 2007; Crew et al., 2010; Dent et al., 2011). Nonpharmacologic strategies that have been suggested to add possible benefit include hot packs, transcutaneous electrical nerve stimulation, massage therapy, acupuncture or acupressure, and psychological interventions. Arthralgias may thus be manageable, decreasing the risk of nonadherence to AI therapy. It is also critical to check serum calcidiol levels in patients who are to receive bisphosphonates or denosumab as part of their therapy or to prevent or treat osteoporosis, so they do not experience severe, life-threatening hypocalcemia. Similarly, this is important for patients who are thought to have bone metastases on x-ray that are subsequently confirmed to be related to osteomalacia.

The issue of screening and supplementation presents a dilemma for APs: Routine calcidiol monitoring is not recommended, and there are no specific recommendations for cancer patients, many of whom are vitamin D deficient. It seems clear that APs can make a case that calcidiol screening is justifiable when identifying vitamin D deficiency, when it may affect treatment outcomes or mortality in certain patients. These include breast cancer patients receiving an AI or tamoxifen, prostate cancer patients on androgen-deprivation therapy, and perhaps patients with colorectal or other cancers (Moyad, 2008).

A second issue is when to reassess serum calcidiol. If a patient is started on a standard dose of vitamin D (e.g., 600–800 IU/day), levels should be rechecked in 3 to 4 months, which is the time it takes for serum calcidiol levels to plateau (Hanley et al., 2010). On the other hand, patients who are given high doses of vitamin D to correct a deficiency should be checked in about 1 month, when peak levels are achieved.

The question of how much supplemental vitamin D to recommend or prescribe, especially for patients with insufficiency, has no clear answer. Hanley et al. (2010) indicated that calcidiol levels increase by 0.28–0.8 ng/mL for each 40 IU of daily vitamin D. For example, the amount of extra vitamin D needed to raise a patient’s calcidiol from 25 to 40 ng/mL would be 750 to 2,143 IU/day. At-risk individuals may need more than the usually accepted doses of vitamin D supplements (e.g., 2,000–5,000 IU/day or 50,000 IU every 1 to 4 weeks) to maintain physiological levels of serum vitamin D and good health. Many endocrinologists prefer their patients to maintain serum vitamin D levels between 30 and 40 ng/mL (75–100 nmol/L).

Another suggestion is to match vitamin D supplementation to calcidiol level (Wimalawansa, 2012). When serum calcidiol is < 10 ng/mL, give 50,000 IU 3 times/wk; for levels of 11 to 20 ng/mL, administer 50,000 IU twice a week; and for 21 to 29 ng/mL, administer 50,000 IU once a week. Each regimen is recommended for 6 to 10 weeks. Once vitamin D levels are normalized, patients should receive maintenance doses of 1,000–2,000 IU per day, 10,000 IU once a week, or 50,000 IU of vitamin D_3_ once a month to prevent reverting to vitamin D deficiency.

Vitamin D toxicity is unlikely to occur with dosages of less than 5,000 IU per day; some studies have confirmed that it is safe to take up to 10,000 IU per day (Wimalawansa, 2012). In fact, vitamin D toxicity is rare and does not occur unless an individual consistently takes at least 40,000 IU/day for several months (Hanley et al., 2010). Manifestations of vitamin D toxicity mirror hypercalcemia: nausea, dehydration, headache, irritability, constipation, hypercalciuria and hyperphosphatemia, and polyuria (Kennel et al., 2010; Wimalawansa, 2012). Most patients with vitamin D toxicity have calcidiol levels > 150 ng/mL.

## Conclusions

In conclusion, APs are vital to maintaining cancer patients’ safety and quality of life by assisting them in maintaining optimal vitamin D levels. In addition, some breast cancer patients may require aggressive and creative symptom management strategies to alleviate arthralgias related to AIMSS
